# Renalase peptides reduce pancreatitis severity in mice

**DOI:** 10.1152/ajpgi.00143.2024

**Published:** 2024-07-16

**Authors:** Thomas R. Kolodecik, Xiaoyu Guo, Christine A. Shugrue, Xiaojia Guo, Gary V. Desir, Li Wen, Fred Gorelick

**Affiliations:** ^1^Veterans Affairs Health Care System, Yale University, New Haven, Connecticut, United States; ^2^Yale School of Medicine, Yale University, New Haven, Connecticut, United States; ^3^State Key Laboratory of Complex, Severe, and Rare Diseases, Institute of Clinical Medicine, Peking Union Medical College Hospital, Chinese Academy of Medical Science & Peking Union Medical College, Beijing, People’s Republic of China; ^4^Center for Biomarker Discovery and Validation, Institute of Clinical Medicine, Peking Union Medical College Hospital, Chinese Academy of Medical Science & Peking Union Medical College, Beijing, People’s Republic of China

**Keywords:** cerulein, endoscopic retrograde cholangiopancreatography, inflammation, renalase, severe pancreatitis

## Abstract

Acute pancreatitis, an acute inflammatory injury of the pancreas, lacks a specific treatment. The circulatory protein renalase is produced by the kidney and other tissues and has potent anti-inflammatory and prosurvival properties. Recombinant renalase can reduce the severity of mild cerulein pancreatitis; the activity is contained in a conserved 20 aa renalase site (RP220). Here, we investigated the therapeutic effects of renalase on pancreatitis using two clinically relevant models of acute pancreatitis. The ability of peptides containing the RP220 site to reduce injury in a 1-day post-endoscopic retrograde cholangiopancreatography (ERCP) and a 2-day severe cerulein induced in mice was examined. The initial dose of renalase peptides was given either prophylactically (before) or therapeutically (after) the initiation of the disease. Samples were collected to determine early pancreatitis responses (tissue edema, plasma amylase, active zymogens) and later histologic tissue injury and inflammatory changes. In both preclinical models, renalase peptides significantly reduced histologic damage associated with pancreatitis, especially inflammation, necrosis, and overall injury. Quantifying inflammation using specific immunohistochemical markers demonstrated that renalase peptides significantly reduced overall bone marrow-derived inflammation and neutrophils and macrophage populations in both models. In the severe cerulein model, administering a renalase peptide with or without pretreatment significantly reduced injury. Pancreatitis and renalase peptide effects appeared to be the same in female and male mice. These studies suggest renalase peptides that retain the anti-inflammatory and prosurvival properties of recombinant renalase can reduce the severity of acute pancreatitis and might be attractive candidates for therapeutic development.

**NEW & NOTEWORTHY** Renalase is a secretory protein. The prosurvival and anti-inflammatory effects of the whole molecule are contained in a 20 aa renalase site (RP220). Systemic treatment with peptides containing this renalase site reduced the severity of post-endoscopic retrograde cholangiopancreatography (ERCP) and severe cerulein pancreatitis in mouse models.

## Graphical abstract

**Figure:**
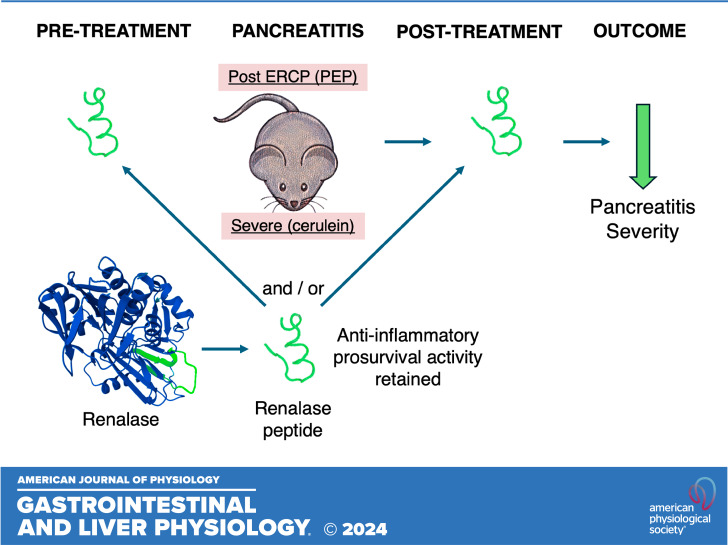

## INTRODUCTION

Acute pancreatitis, an inflammatory disease of the pancreas, is one of the most common reasons for hospital admission with gastrointestinal illness. The most frequent causes of acute pancreatitis include gallstones, alcohol, cigarette smoking, endoscopic retrograde cholangiopancreatography (ERCP), and surgical complications (1, 2). There are no specific pharmacologic treatments for acute pancreatitis, and in most cases, fluid replacement and pain management are the only interventions. In post-ERCP-acute pancreatitis (PEP), placing a temporary pancreatic duct stent or prophylactically administering rectal nonsteroidal anti-inflammatory drugs such as indomethacin (3) can reduce the incidence and severity of pancreatitis.

Preclinical studies of acute pancreatitis in animal models and clinical acute pancreatitis demonstrate that exuberant inflammatory responses are a disease hallmark and relate to its severity. Several classes of anti-inflammatory agents have been found to reduce acute pancreatitis severity in preclinical models. Examples include TNFα inhibition (4), blockers of cell calcium entry, Orai1 inhibitors (5, 6), and an anti-inflammatory drug used to treat idiopathic pulmonary fibrosis, pirfenidone (7). The latter two agents can reduce acute inflammatory responses in whole animal studies, tissue, and cellular preparations.

Renalase is a 37-kDa secretory protein found in plasma and tissue. The renalase-1 isoform is the most highly expressed in humans and mice, though multiple splice variants have been identified. Renalase is predominately produced by the proximal tubule cells of the kidney but is also made by other cell types and tissues. During acute experimental and clinical pancreatitis, plasma levels of renalase can significantly decrease (8, 9). In models of acute injury, including to the kidney, heart, and pancreas, full-length recombinant renalase treatment has dramatic protective effects, sometimes showing benefit when given after the onset of injury (8, 10, 11).

**Figure 1.**
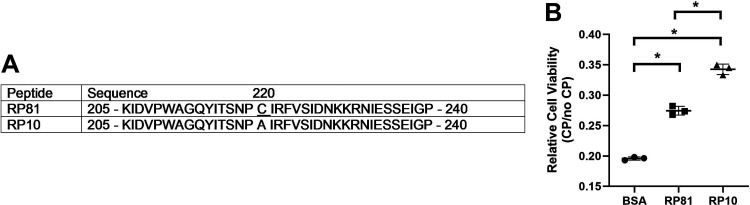
Renalase peptides. *A*: sequences of peptides used in our studies. The sequence of both peptides corresponds to amino acids 205–240 of human renalase-1 except for a single amino acid substitution in RP10 of alanine for cysteine at position 220. *B*: TKPTS cells were pretreated with renalase peptides RP81 (12.5 mM) or RP10 (12.5 mM) for 24 h and followed by treatment with either vehicle or cisplatin and cell viability was assessed by WST assay. *P < 0.05, *n* = 3 for all groups.

We reported that renalase’s prosurvival and anti-inflammatory activities are contained in a 20 aa site (RP220), which is highly conserved among known splice variants and species (10). The RP220 peptide binds to a plasma-membrane renalase receptor and effector, the plasma membrane calcium ATPase 4B (PMCA4B) (12). Given the cost and difficulty in producing full-length renalase for therapeutic use, we questioned if peptides containing the RP220 site could reduce pancreatitis severity. We have shown that mesoscale nanoparticles targeting the kidney loaded with a renalase peptide (RP81: Fig. 1*A*) containing the RP220 site can significantly reduce the severity of cisplatin-induced renal injury (13). The limitations of this study included limited relevance to other forms of acute injury and the need to deliver the renalase peptide directly to the kidney to see a therapeutic effect (13). Here, we examined the effectiveness of treatments with RP81 and a more soluble related-renalase peptide, RP10 (Fig. 1*A*), given systemically in murine models of post-ERCP pancreatitis (PEP) and severe cerulein acute pancreatitis to reduce injury. In both models, the renalase peptides significantly decreased inflammation and the associated cellular injury and tissue damage. Our data suggest that renalase peptides, which retain their anti-inflammatory and prosurvival activities, could be attractive therapeutic candidates for reducing acute pancreatitis severity.

## MATERIALS AND METHODS

### Ethics Statement

All experiments and procedures using animals in West Haven, CT, were approved by the Veterans Administration Institutional Animal Care and Use Committee, West Haven, CT (Veterans Administration Public Health and Safety, Office of Laboratory Animal Welfare Assurance Number A4363-01). All experiments and procedures using animals in Shanghai, People’s Republic of China, were approved by the Animal Ethics Committee of Shanghai General Hospital (2019-A019-01). All authors had access to the study data and had reviewed and approved the final manuscript.

### Renalase Peptides

The anti-inflammatory and prosurvival activity of renalase is contained in a site (human renalase-1 aa 220 to 239) highly conserved among renalase splice variants and species (10). Renalase peptides RP10 and RP81 were included in this site and extended to include aa 205 to 240 of human renalase-1 to promote their stability. It was observed that the solubility of the RP81 peptide could be enhanced by replacing its cysteine residue (aa16) with an alanine. This renalase peptide was designated RP10. RP10 was kindly provided by Bessor Pharma LLC and synthesized by LifeTein (Somerset, NJ). RP81, used in People’s Republic of China, was synthesized by GL Biochem Ltd. (Shanghai, PR China). Peptides were dissolved in half of the final volume of sterile water. Once dissolved, they were diluted to the final volume with 300 mM sterile saline (150 mM final). The peptide injection volume was 200 µL subcutaneously (sc). Preliminary studies in the severe cerulein pancreatitis model suggested a dose of 20 mg/kg of the renalase peptides provided maximal benefit.

### Cell Culture and Cell Viability Assay

To test the prosurvival effects of these renalase peptides in cultured cells, we used the human proximal tubule cell line HK-2 (ATCC No. CRL-2190, Manassas, VA). HK-2 cells at 70% confluence were pretreated with 12.5 µM RP81(CSBio, Menlo Park, CA) or RP10 for 24 h, followed by treatment with either vehicle (saline) or 25 µM cisplatin (Sigma, Burlington, MA) for 24 h. WST-1 reagent (Takara Bio, Inc, Kusatsu, Shiga, Japan; Cat. No. MK400) was added to cells at 1:10 dilution and incubated for 40 min. Absorbance was then read at 450 nm (Formazan) and 600 nm (Reference). Formazan production was corrected for background by subtracting the reference value (*A*450 − *A*600 = *A* of Formazan). Relative cell viability is the value of the cisplatin-treated cells divided by that of the nontreated cell (CP/non-CP).

### Models of Pancreatitis and Treatment with Renalase Peptides

#### Post-ERCP model.

**Figure 2.**
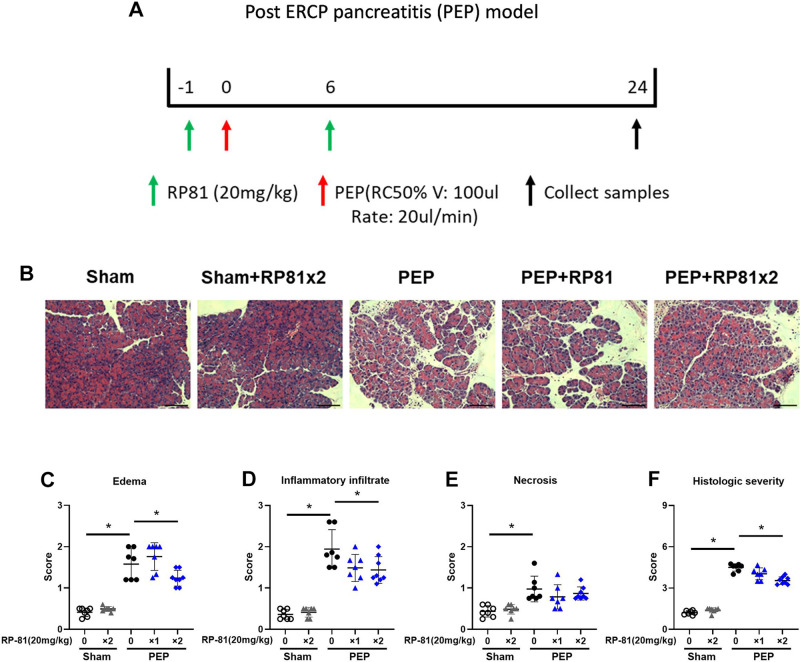
Renalase peptide RP81 treatment reduces histological damage in post-endoscopic retrograde cholangiopancreatography (ERCP) pancreatitis in male mice. *A*: schematic for administering RP81 (20 mg/kg) and the induction of post-ERCP pancreatitis (PEP). *B*: representative hematoxylin and eosin (H&E) images of the pancreas. Magnification ×200; scale bar: 100 μm). Scores for edema (*C*), inflammation (*D*), necrosis (*E*), and total histological score (*F*). Data: individual values and means ± SD (*n* = 7 or 8/group). *P < 0.05 between indicated groups.

Postendoscopic retrograde cholangiopancreatography (ERCP) pancreatitis (PEP) shown in Fig. 2*A* was induced as detailed by Jin et al. (14) and Wen et al. (15). Briefly, 100 µL of iodiproamine (Ultravist 370, Bayer Healthcare Company Ltd., Wan Chai, HK, PR China) was infused retrograde into the biliopancreatic duct at a rate of 20 µL/min for 5 min. Mice from the sham group received laparotomy only. The renalase peptide (RP81, given 20 mg/kg sc) was injected 1 h before the induction (1×) or with an additional dose 6 h after (2×) (Fig. 2*A*). Samples of serum and tissue were collected 24 h after the induction to determine severity.

#### Severe cerulein model.

The model for prophylactic RP10 treatment of acute cerulein pancreatitis is shown in Fig. 6*A*. Severe cerulein pancreatitis was induced as described with slight modification (7). Briefly, male or female C57/BL6 mice weighing ∼25 g were given eight hourly intraperitoneal (ip) injections of either cerulein (50 µg/kg) or saline (control) in a volume of 200 µL for two consecutive days. Animals given prophylactic RP10 treatment also received three injections of either a renalase peptide RP10 (10 or 20 mg/kg sc) or normal saline. The first dose was given 1 h before inducing pancreatitis, followed by a dose 6 h later and 1 h before giving cerulein on *day 2* (Fig. 2*A*). Mice given therapeutic RP10 treatment (after inducing pancreatitis), as described in Fig. 12*A*, did not receive the first prophylactic dose of RP10 but were given the second and third doses. Mice were euthanized, and tissues were collected 1 h after the last cerulein injection on *day 2*. Renalase peptide treatment protocols were selected based on preliminary testing of various doses and administration frequencies in the severe cerulein model.

### Immunohistochemistry and Histologic Staining

Immunohistochemistry was performed as described previously (16). In brief, pancreatic tissue sections (5 μm) from formalin-fixed and paraffin-embedded tissues were deparaffinized and hydrated. Antigen retrieval was done by exposing samples on glass slides to citrate buffer (10 mM citric acid pH 6) for 20 min in a steamer. Nonspecific labeling was reduced with BLOXALL blocking solution (Vector Laboratories, Burlingame, CA) for 15 min followed by 1 h with 2.5% normal serum (Vector Laboratories; species dependent on that of secondary antibody). Primary antibodies were selected based on vendor documentation of specificity. The following primary antibodies were diluted with Tris-buffered saline (TBS) + 0.1% Tween-20 as indicated and incubated overnight at 4°C in a humidified chamber CD45 (1:100, Cell Signaling Technologies, Danvers, MA; 70257s), Arginase-1 (1:100, Cell Signaling Technologies; 93668s), Ly-6B.2 (1:100, BioRad, Hercules, CA; MCA154115), and CD86(1:400, Cell Signaling Technologies; 19589-s). Primary antibodies of the appropriate species were detected using ImmPRESS peroxidase-anti IgG (Vector Laboratories). Color was developed using a DAB substrate kit and counterstained with methyl green (Vector Laboratories). The DAB reaction was performed for a fixed time. Coverslips were mounted using Vectamount (Vector Laboratories). Nonspecific labeling was evaluated for each tissue by labeling in the absence of primary antibodies. Images of 10 different unselected fields were acquired in each treatment group. Rarely, images from fewer than 10 field images were acquired because of limited tissue size or damage. All images were captured at ×400 using a Spot RT3 camera (Spot Imaging Solutions, Sterling Heights, MI), and imaging software, and the percentage area of positive labeling was quantitated for each image using ImageJ as described previously (17).

Sections for histologic evaluation were stained with hematoxylin and eosin (H&E) and examined by light microscopy at ×400 magnification. Histologic markers of pancreatitis severity were scored blinded using described criteria (7, 18).

### Amylase Assay

Plasma amylase content was measured according to the manufacturer’s instructions (Phaebadas kit, Magle Life Sciences, Lund, Sweden).

### Zymogen Activation Assay

Pancreatic tissue was homogenized in assay buffer [50 mM Tris (pH 8.1), 150 mM NaCl, 1 mM CaCl_2_] and centrifuged at 500 *g* for 5 min to generate a postnuclear supernatant (PNS). As described previously, the PNS was assayed for zymogen activity using fluorogenic substrates (8). Briefly, 50 µL of enzyme-substrate (40 mM final) (chymotrypsin; Calbiochem, a division of EMD Chemicals Inc, Gibbstown, NJ and trypsin; Peptides International, Louisville, KY) was added to each well containing 20–50 µL of sample and 400–430 µL of the assay reagents for a total volume in the well of 500 µL. The plate was then read using a fluorometric plate reader (Flx800, BioTek Instruments, Winooski, VT) at excitation wavelength 380 nm and emission 440 nm for 20 measurements over 10 min. The slope of the resulting line, which represents the enzyme activity of the homogenate, was normalized to total protein content as determined by Pierce 660 protein assay according to the manufacturer’s directions (Thermo Scientific, Waltham, MA).

### Measurement of Tissue Water Content (Edema)

Pancreatic tissue was harvested, adherent fat was removed, and its wet weight was determined. The tissues were dried at 60°C for at least 48 h and reweighed. Edema was expressed as percent water content (wet weight − dry weight/wet weight × 100).

### Statistical Analysis

All statistical analyses were performed using Prism-10 software (GraphPad Software). Data represent the means ± standard deviation (SD) of at least three individuals unless otherwise noted. An unpaired *t* test with Welch’s correction was used, and a *P* value of <0.05 was assigned significance. Data were assessed for outliers using Prism software-10 (GraphPad Software).

## RESULTS

### Renalase Peptide Treatment Increases Cell Viability in Cultured Cells

To address the issues of solubility, aggregation, and stability of RP81 in our experiments, we generated multiple amino acid variants of the peptide. We have found that cellular prosurvival responses to cisplatin-induced injury for renalase peptides correspond to their effectiveness in vivo. When the renalase peptide RP10 was tested in HK-2 cells challenged with cisplatin, it showed a greater increase in cell survival compared with other renalase-related peptides, including RP81 (Fig. 1*B*), and it was selected for subsequent testing.

### Prophylactic Treatment with RP81 in Post-ERCP Pancreatitis Is Effective

**Figure 3.**
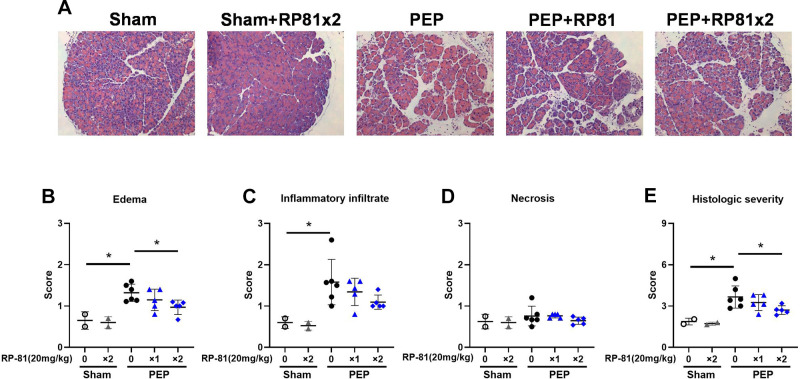
Renalase peptide RP81 treatment reduces histological damage in post-endoscopic retrograde cholangiopancreatography (ERCP) pancreatitis in female mice. *A*: representative hematoxylin and eosin (H&E) images of the pancreas (magnification: ×200, scale bar: 100 μm). Scores for edema (*B*), inflammation (*C*), necrosis (*D*), and total histological score (*E*). Data: individual values and means ± SD (*n* = 7 or 8/group). *P < 0.05 compared with the sham group. *P < 0.05 between indicated groups. PEP, post-ERCP pancreatitis.

Post-ERCP pancreatitis (PEP) induced by retrograde infusion of radiocontrast at high pressure (14, 15) was used to determine if pretreatment with the renalase peptide RP81 containing the RP220 area was histologically protective (Fig. 2). Histologic levels of edema, inflammatory infiltration, and necrosis were significantly increased in PEP (Fig. 2*B*). Treatment with RP81 significantly decreased histologic injury as assessed by edema, inflammation, and the histologic composite score (Fig. 2, *C–F*, respectively). The two-dose regimen was slightly more effective than pretreatment alone and was used in subsequent studies. Similar responses were observed for pancreatitis severity and the effectiveness of RP81 in male (Fig. 2) and female mice (Fig. 3).

The inflammatory cell responses in acute pancreatitis involve many cell types and are time dependent (19, 20). Since interest in the injury responses has focused on bone marrow-derived cell types in early disease, especially neutrophils and macrophages, we quantified these populations using immunohistochemistry.

**Figure 4.**
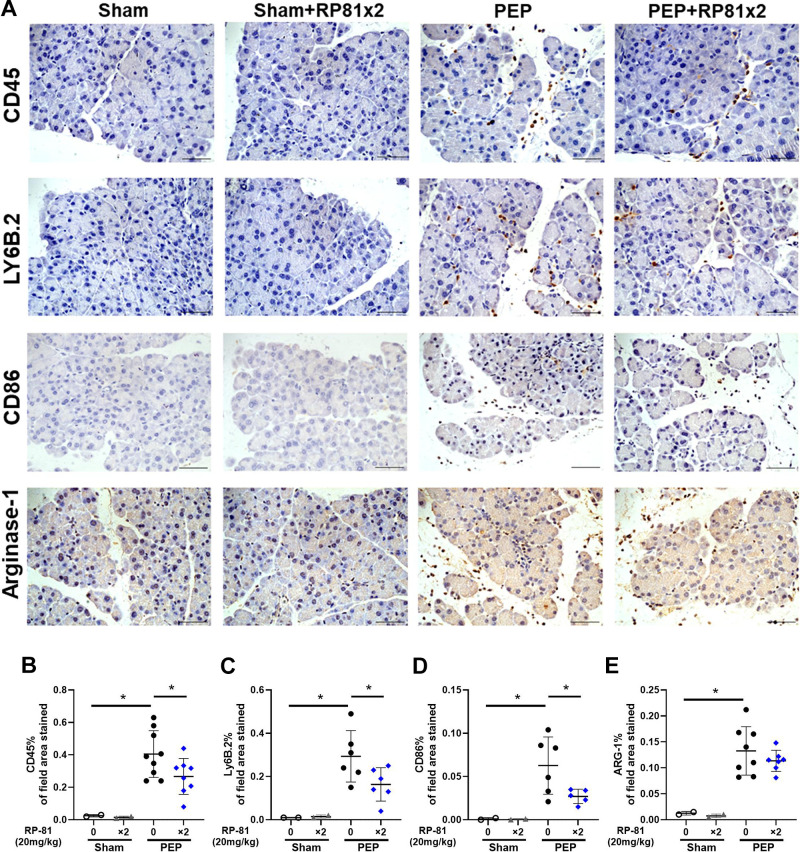
Renalase peptide RP81 treatment inhibits inflammatory cell infiltration in post-endoscopic retrograde cholangiopancreatography (ERCP) pancreatitis in male mice. *A*: representative pancreatic images of immunostaining for CD45, a general leukocyte marker, LY6B.2, a neutrophils marker, CD86, a M1 macrophage marker and arginase-1, a M2 macrophage marker. Magnification ×400; scale bar: 50 μm. Quantification of CD45 (*B*), Ly6B.2 (*C*), CD86 (*D*), and ARG-1 (*E*) staining using ImageJ. Values were plotted as percentage of the total area positively stained. Data: means ± SD (*n* = 3–9/ group). *P < 0.05 between indicated groups. PEP, post-ERCP pancreatitis.

**Figure 5.**
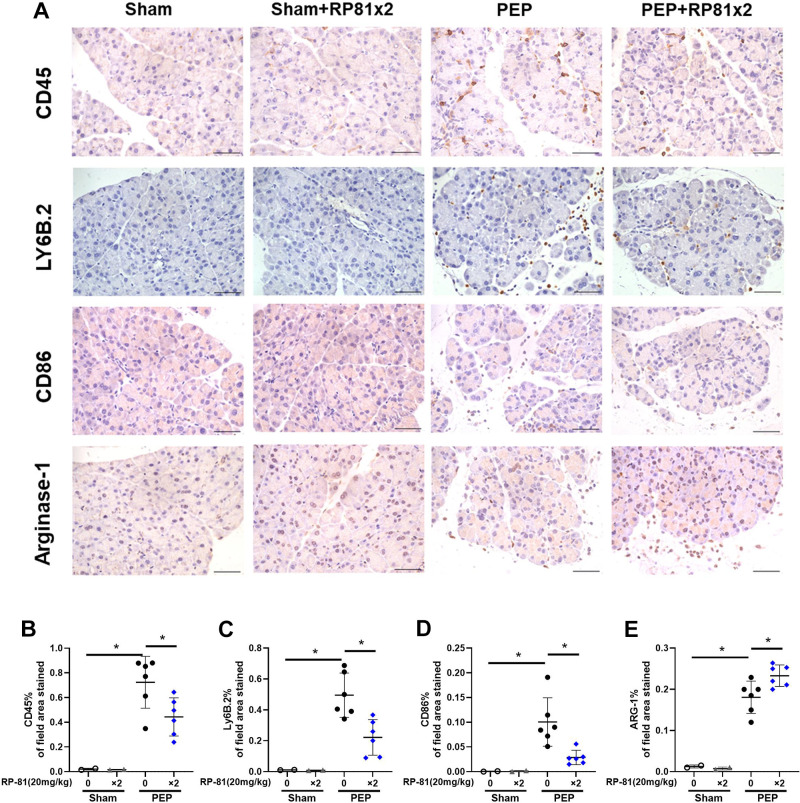
Renalase peptide RP81 treatment inhibits inflammatory cell infiltration in post-endoscopic retrograde cholangiopancreatography (ERCP) pancreatitis in female mice. *A*: representative pancreatic images of immunostaining for CD45, a general leukocyte marker, LY6B.2, a neutrophil marker, CD86, a M1 macrophage marker, and arginase-1, a M2 macrophage marker. Magnification: ×400, scale bar: 50 μm. Quantification of CD45 (*B*), Ly6B.2 (*C*), CD86 (*D*), and ARG-1 (*E*) staining using ImageJ. Values were plotted as percentage of the total area. *P < 0.05 between indicated groups.

Few inflammatory cells were present in pancreatic tissues from sham-operated controls (Fig. 4*A*) or sham-operated mice treated with peptide alone (Fig. 4*A*). However, overall bone-marrow-derived inflammatory cell (CD45) labeling appeared to be dramatically increased during PEP (Fig. 4*A*) and was seen in the edematous interstitial spaces and between acini (Fig. 4*A*). Quantitation confirmed the CD45 increase, which was significantly reduced by renalase peptide RP81 treatment (Fig. 4*B*). Similar effects were observed for other inflammatory cell markers, including Ly6B.2 for neutrophils, CD86 for M1 macrophages, and Arginase-1 for M2 macrophages (Fig. 4, *C–E*, respectively). The effects of PEP and RP81 in female mice (Fig. 5, *A–E*) were similar to those in male mice. The data suggest that the renalase peptide RP81 treatment can significantly reduce both neutrophilic and macrophage inflammation in a murine model of PEP. Having demonstrated the effectiveness of a renalase peptide in mild pancreatitis, we next asked if RP10 could reduce injury in a model of severe cerulein pancreatitis.

### Prophylactic Treatment with RP10 in Severe Cerulein Pancreatitis Is Effective

**Figure 6.**
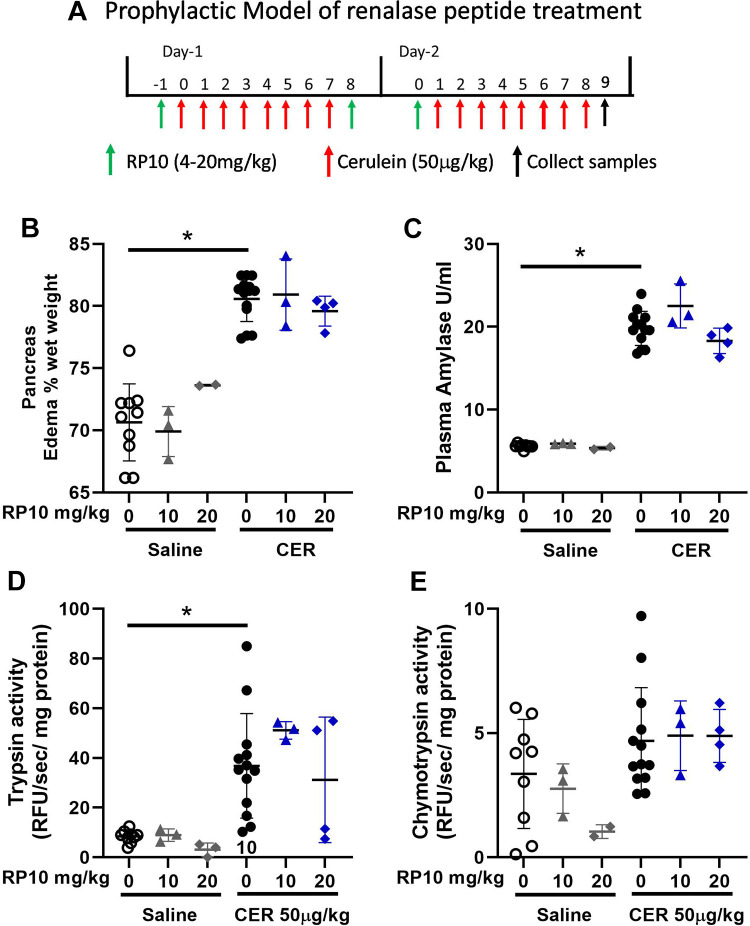
Renalase peptide RP10 pretreatment may have little effect on select acute pancreatitis markers in male mice. *A*: diagram of severe cerulein (CER) pancreatitis induction and renalase treatment. Samples were collected and assayed as described in materials and methods. Tissue edema (dry/wet weight, *B*), plasma amylase (*C*), trypsinogen activation (*D*), and chymotrypsinogen activation (*E*). Graphs include individual values and the means ± SD. *n* = 3 or more for all treatment groups except for 20 mg/kg RP10 alone which was *n* = 2. *P < 0.05 compared with saline alone.

**Figure 7.**
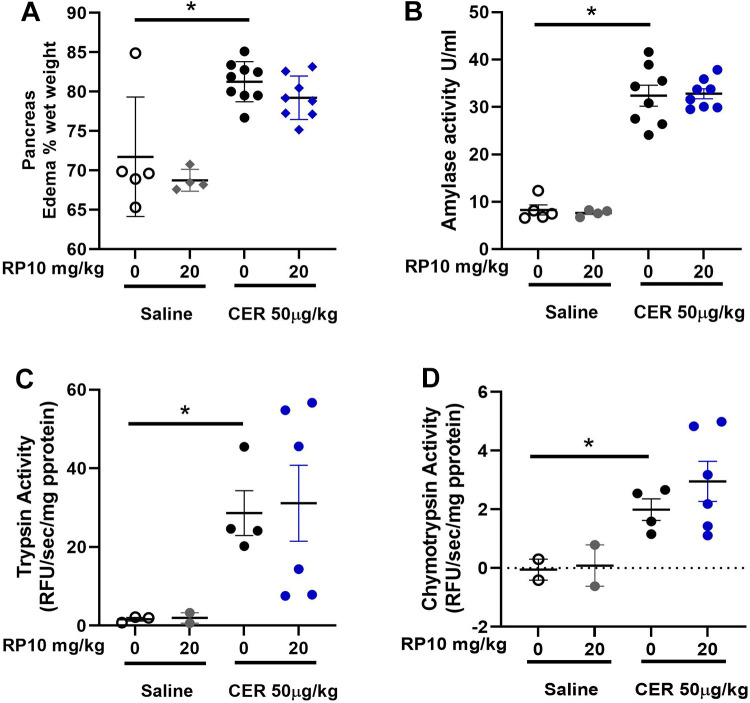
Renalase peptide RP10 pretreatment may have little effect on select acute pancreatitis markers in female mice. Samples were collected and assayed as described in materials and methods. Tissue edema (dry/wet weight, *A*), plasma amylase (*B*), trypsinogen activation (*C*), and chymotrypsinogen activation (*D*). Graphs include individual values and the means ± SD. *n* = 3 or more for all treatment groups except for 20 mg/kg RP10 alone for trypsin and chymotrypsin which was *n* = 2. *P < 0.05 compared with saline alone. CER, cerulein.

Induction of pancreatitis after 33 h was confirmed using biochemical, histologic, and inflammatory markers. Cerulein treatment resulted in significant increases in pancreatic water content (edema) (Fig. 6*B*), plasma amylase approximately fourfold (Fig. 6*C*), and trypsin activity (Fig. 6*D*). Though these early markers of pancreatitis tended to decrease at the higher dose of the renalase peptide RP10, the changes were not statistically significant. There were no significant changes in chymotrypsin activities with cerulein treatment or the addition of RP10 (Fig. 6*E*). The effects of cerulein and RP10 in female mice (Fig. 7, *A–D*) were similar to those in male mice.

**Figure 8.**
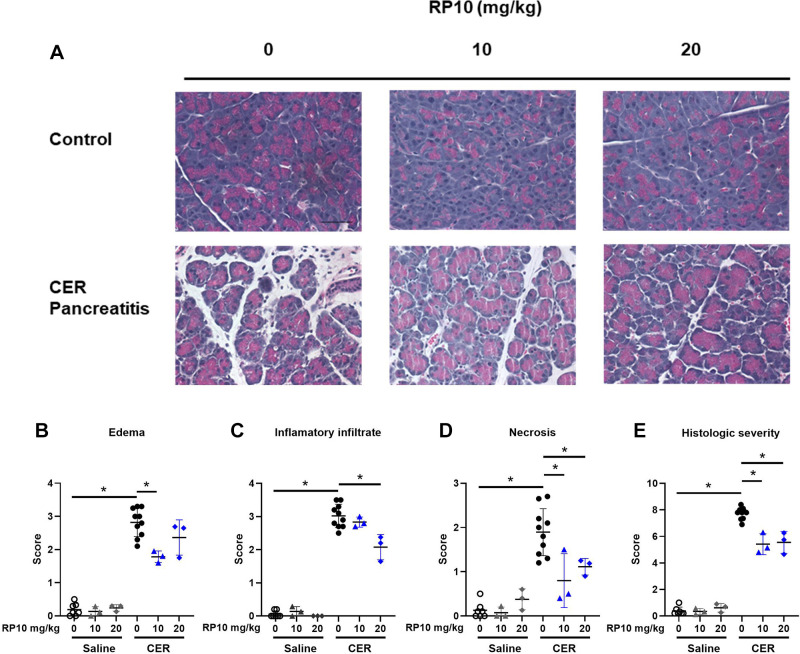
Renalase peptide RP10 pretreatment decreases histologic damage in severe cerulein (CER) pancreatitis in male mice. After inducing severe CER and treatment with or without a renalase peptide (33 h), tissues were fixed in buffered formalin and hematoxylin and eosin (H&E) stained. *A*: representative images of treatment groups. Random photographs of ten different fields were taken at ×400 magnification. Each image was scored blinded for histologic markers of pancreatitis based on published criteria, including edema (*B*), inflammatory cell infiltration (*C*), and necrosis (*D*). Scores were summed within each category and presented as total histologic score (*E*). Data: individual values and means ± SD. *n* = 3 or more for all treatment groups. *P < 0.05 between indicated groups.

**Figure 9.**
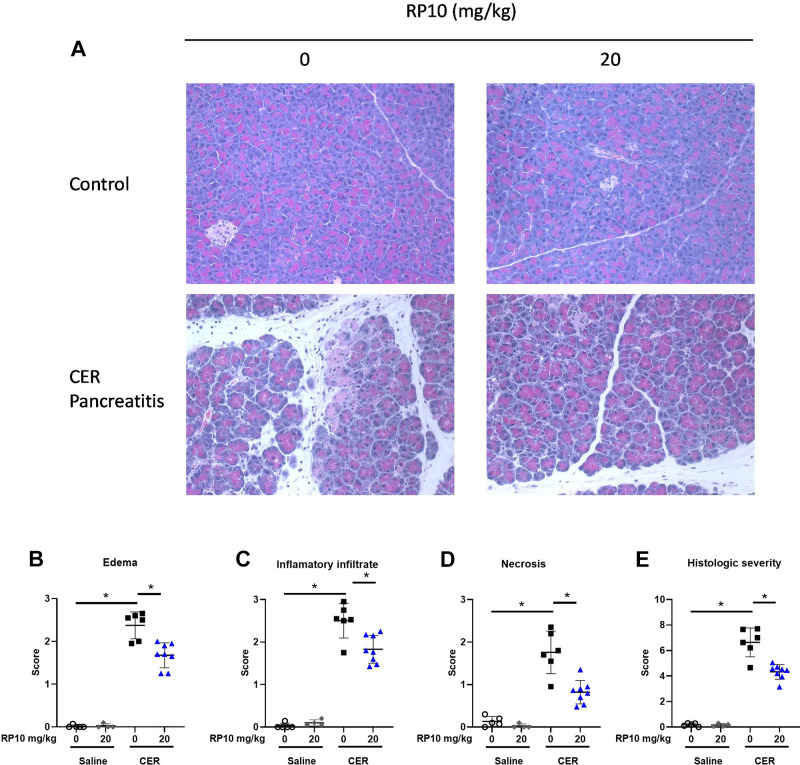
Renalase peptide RP10 pretreatment decreases histologic damage in severe cerulein (CER) pancreatitis in female mice. After inducing severe cerulein and treatment with or without a renalase peptide (33 h), tissues were fixed in buffered formalin and hematoxylin and eosin (H&E) stained. *A*: representative images of treatment groups. Random photographs of 10 different fields were taken at ×400 magnification. Each image was scored blinded for histologic markers of pancreatitis based on published criteria, including edema (*B*), inflammatory cell infiltration (*C*), and necrosis (*D*). Scores were summed within each category and presented as total histologic score (*E*). Data: individual values and means ± SD. *n* = 3 or more for all treatment groups. *P < 0.05 between indicated groups.

Pancreatic histology showed acute pancreatitis 33 h after induction (Fig. 8*A*). Blinded scoring showed significant increases over baseline edema, immune cell infiltration, necrosis, and a composite histologic score (Fig. 8, *B–E*). Treatment with a renalase peptide RP10 did not change baseline histology, but it appeared to reduce histologic inflammation in severe cerulein-induced pancreatitis. The effects of the renalase peptide on inflammation (Fig. 8*C*) tended to be dose dependent, with the higher dose significantly reducing histologic injury (Fig. 8, *B–E*). Similar effects were found in female mice (Fig. 9, *A–E*). These findings suggest that treatment with a renalase peptide containing the RP220 site can reduce the severity of acute pancreatitis measured histologically.

**Figure 10.**
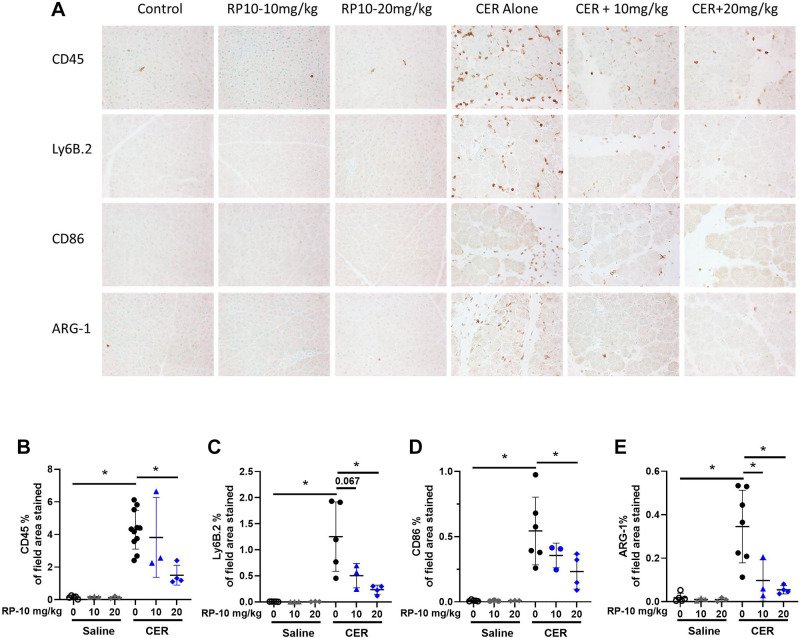
Renalase peptide RP10 pretreatment inhibits inflammatory cell infiltration in cerulein (CER)-stimulated severe pancreatitis in male mice. CER pancreatitis was induced in animals with and without RP10 treatment as described earlier. Tissues were fixed in buffered formalin and labeled for specific immune cell markers using immunohistochemistry. *A*: representative images of each treatment group. Slides were examined, and 10 random fields were randomly imaged at ×400 magnification. Labeling was quantitated using ImageJ. Values were plotted as percentage of the total area positively stained. Markers included CD45 (general leukocytes, *B*), Ly6B.2 (neutrophils, *C*), CD86 (M1 macrophages, *D*), and Arginase1 (M2 macrophages, *E*). Data: individual values and means ± SD. *n* = 3 or more for all treatment groups. *P < 0.05 between indicated groups. Where comparisons were close to significance actual *P* values are given.

**Figure 11.**
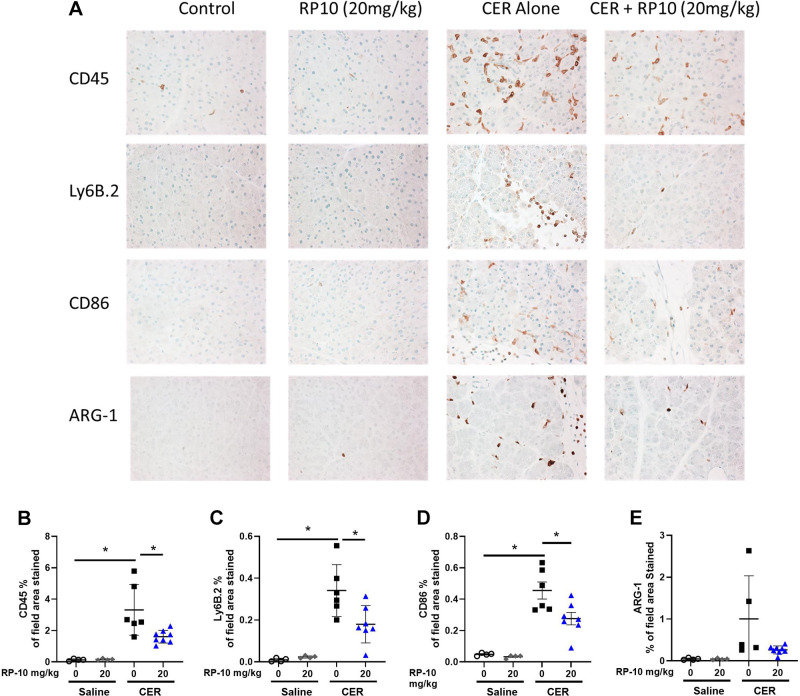
Renalase peptide RP10 pretreatment inhibits inflammatory cell infiltration in cerulein (CER)-stimulated severe pancreatitis in female mice. CER pancreatitis was induced in animals with and without RP10 treatment as described earlier. Tissues were fixed in buffered formalin and labeled for specific immune cell markers using immunohistochemistry. *A*: representative images of each treatment groups. Slides were examined, and ten random fields were randomly imaged at ×400 magnification. Labeling was quantitated using ImageJ. Values were plotted as percentage of total area positively stained. Markers included CD45 (general leukocytes, *B*), Ly6B.2 (neutrophils, *C*), CD86 (M1 macrophages, *D*), and Arginase1 (M2 macrophages, *E*). Data: individual values and means ± SD. *n* = 3 or more for all treatment groups. *P < 0.05 between indicated groups. Near-significant actual *P* values are given.

Inflammatory cell populations were quantified by quantitative immunocytochemistry using markers for bone marrow-derived inflammatory cells (overall, neutrophils and M1 and M2-like macrophages). Few inflammatory cells are present in either saline-treated control tissues or controls given either dose of the renalase peptide (Fig. 10*A*). Pancreatic labeling for CD45-positive cells was dramatically increased after cerulein treatment and seen in both the interstitial spaces and between acini (Fig. 10*A*). CD45 labeling appeared to decrease in tissues from animals treated with renalase peptide in a dose-dependent manner (Fig. 10*A*). This decrease was confirmed by quantifying CD45 labeling (Fig. 10*B*). Similar effects were observed for other inflammatory markers, including Ly6B.2 (Fig. 10, *A* and *C*: neutrophils), CD86 (Fig. 10, *A* and *D*: M1 macrophages), and Arg1 (Fig. 10, *A* and *E*: M2 macrophages). Data were obtained in female mice for pancreatitis responses, and the effects of RP10 mice were similar to those of males (Fig. 11, *A–D*). An exception was the lack of significant changes in Arg1-labeled cells in female mice, likely due to the high variability in the cerulein (CER) alone group (Fig. 11, *A* and *D*). The data suggest that severe cerulein pancreatitis is associated with a broad increase in pancreatic infiltration of neutrophils and macrophages, which is significantly reduced by renalase peptide treatment. These effects were most apparent at the highest dose of the renalase peptide (20 mg/kg).

### Therapeutic Treatment with RP10 in Severe Pancreatitis Is Effective

**Figure 12.**
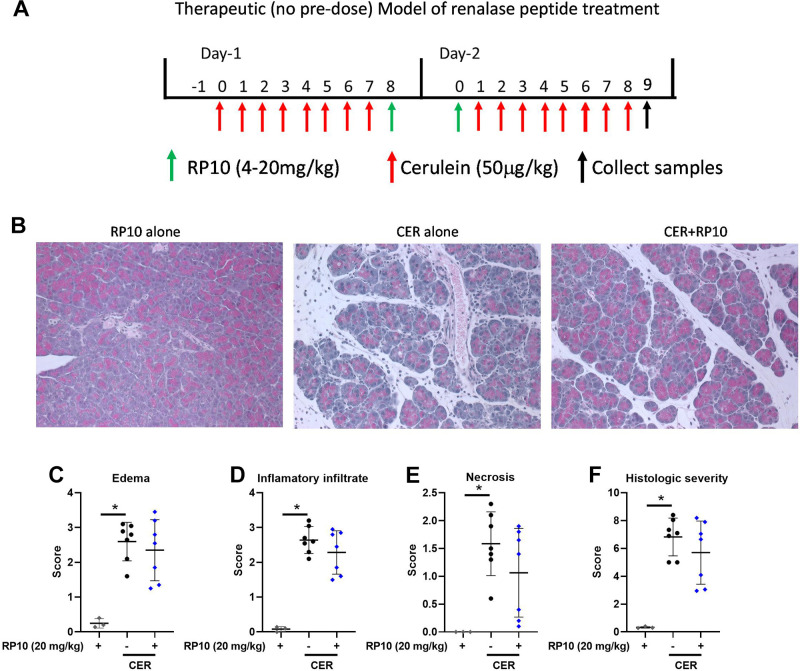
Therapeutic treatment with renalase peptide RP10 tends to decrease histologic injury in severe cerulein (CER) pancreatitis. *A*: diagram of severe CER pancreatitis induction and therapeutic renalase treatment. Samples were collected and assayed as described in materials and methods (33 h), tissues were fixed in buffered formalin and hematoxylin and eosin (H&E) stained. *B*: representative images of treatment groups. Random photographs of 10 different fields were taken at ×400 magnification. Each image was scored blinded for histologic markers of pancreatitis based on published criteria, including edema (*C*), inflammatory cell infiltration (D), and necrosis (*E*). Scores were summed within each category and presented as total histologic score (*F*). Data: individual values and means ± SD. *n* = 3 or more for all treatment groups. *P < 0.05 between indicated groups.

**Figure 13.**
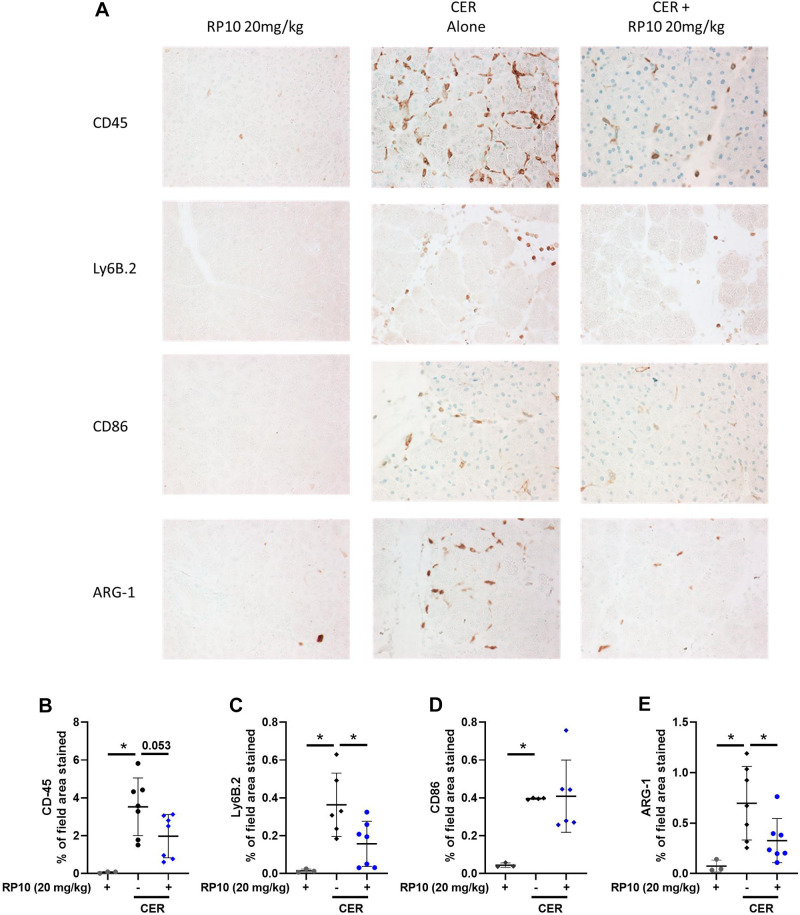
Therapeutic treatment with renalase peptide RP10 decreases select inflammatory responses in severe cerulein (CER) pancreatitis. Tissues were fixed in buffered formalin and labeled for specific immune cell markers using immunohistochemistry. *A*: representative images of each treatment groups. Slides were examined, and 10 random fields were randomly imaged at ×400 magnification. Labeling was quantitated using ImageJ. Values were plotted as percentage of the total area positively stained. Markers included CD45 (general leukocyte, *B*), Ly6B.2 (neutrophils, *C*) CD86 (M1 macrophages, *D*), and Arginase1 (M2 macrophages, *E*). Data: individual values and means ± SD. *n* = 3 or more for all treatment groups. *P < 0.05 between indicated groups. Where comparisons were near significance, *P* values between indicated groups are given.

In a limited number of studies done in male mice, we omitted the first preinduction dose of the RP10 (20 mg/kg) and only gave doses at 8 h and 24 h. Pancreatic damage and inflammation, as determined by histology, tended to decrease with RP10 treatment but did not reach statistical significance (Fig. 12, *B–F*). Inflammatory cell subtypes were detected by immunohistochemistry (Fig. 13*A*). Labeling appeared to decrease with renalase peptide treatment. Quantification of the immune cell infiltrates confirmed a significant decrease for Ly6B.2 positive cells (Fig. 13*C*) and Arg-1 (Fig. 13*E*) but not CD86 (Fig. 13*D*). CD45 infiltration appeared to decrease but did not reach significance (Fig. 13*B*). These studies suggest that the RP10 renalase peptide can reduce the severity of acute pancreatitis when less is given and when administered long after induction of the disease.

## DISCUSSION

Specific treatments that reduce acute pancreatitis injury are lacking. Here, we provide the first evidence that systemic administration of a peptide containing a site in human renalase 1 reduces the severity of pancreatitis in two preclinical mouse models (8). This conserved 20 aa site of renalase retains the anti-inflammatory and prosurvival properties of renalase and has been designated RP220 (10). Molecular modeling (AlphaFold, EMBL-EBI, Hinxton, Cambridgeshire, UK) of the crystal structure (RCSB-PDB No. 3QJ4) (21) suggests these renalase residues are stabilized on the surface of renalase and could bind to its receptor the plasma-membrane calcium ATPase, PCMC4b. RP81 has been shown to prevent injury in other systems but can be difficult to solubilize. We have shown that only when RP81 is encapsulated and targeted to the kidney and not given systemically can it reduce acute renal injury (13).

Because the solubility of RP81 was sometimes problematic, we replaced cysteine residue at aa 16 of the RP81 peptide with an alanine. The new peptide, RP10, had significantly greater survival effects in TKPTS cells treated with the toxin cisplatin than RP81. Here, we used both RP81 and RP10 peptides in vivo and observed that when administered untargeted, they reduced acute pancreatitis severity in two preclinical mouse models of PEP (RP81) and severe cerulein (RP10), which differ in their induction mechanisms.

In the PEP model, significant improvements in histologic injury were seen with two doses of the peptide (pretreatment and 6 h after initiation). When only the pretreatment dose was given, the histology appeared to improve, but the changes did not reach statistical significance. With respect to inflammatory cell types, the two-dose regimen significantly reduced overall bone marrow-derived inflammatory cells (CD45), as well as neutrophils and inflammatory M1-like macrophages but not anti-inflammatory M2-like macrophages.

We found that in our severe pancreatitis model, prophylactic treatment with the renalase peptide (10 mg and 20 mg/kg) significantly reduced histologic injury (edema, inflammation, necrosis, overall histologic severity) and, using immunologic markers, overall inflammation and inflammation by neutrophils and macrophages. The responses to the 10 mg/kg and 20 mg/kg doses were similar, with a tendency for the higher dose to be more effective. Studies using a therapeutic protocol that omitted the first dose (pretreatment) of RP10 to reflect a common clinical scenario also showed significant decreases in inflammation. Thus, the renalase peptide RP10 can reduce severe pancreatitis responses when given systemically, benefits male and female mice, and is protective when given in a therapeutic format.

Sex-dependent differences in the risk of developing PEP and experiencing more severe disease are recognized for young women over young men (22, 23). Female sex has also been suggested as a dominant risk factor for PEP in older people (24). In this study, we assessed pancreatitis severity and therapeutic responses to determine if there were any sex-based differences in responses. In the PEP model, histologic edema, inflammation, and overall histology were very similar, with the females tending to be slightly less severe. In the male mice, we saw a significant increase in necrosis, which was not seen in females. The baseline in the males was lower than in the females, possibly due to the limited number of untreated sham females. There were no definitive differences in overall inflammation or inflammatory cell subtypes between male and female mice. As was seen in the PEP model, the severe cerulein pancreatitis model showed similar histologic changes in both sexes, with the females again showing slightly less injury. We observed a difference in inflammatory cell infiltration, where neutrophilic infiltration appeared to be greater in male mice, whereas M2 macrophages were greater in female mice. Little, if any, differences were observed in responses to renalase peptide treatment between male and female mice. Overall, the pancreatitis and responses to treatment were very similar for male and female mice in both models.

The cellular targets of renalase in acute injury remain unclear. We reported that it reduces injury in isolated groups of acinar cells (8), but its effects on other critical cell populations, especially those regulating innate immune responses, are largely unknown. We have shown that renalase can reduce inflammasome activation in macrophages (25), an important innate immune response in acute pancreatitis. However, the importance of this response, the effects of renalase peptides on other members of the innate immune response, and whether the actions are direct or indirect remain unclear.

Regulation of plasma membrane calcium flux in the pancreatic acinar cell is critical to the pathology of acute pancreatitis. Roles for calcium entry and plasma membrane calcium exporters in acute pancreatitis have been shown, including calcium entry through the Orai1 and Piezo1/TRPV4 transporters (6, 26). Renalase can act through its receptor, the plasma-membrane calcium exporter, PMCA4B (12), which is found on pancreatic acinar cells and modulates cellular injury (8). Others reported that this calcium exporter can mediate pancreatitis responses (27) and contribute to insulin’s beneficial effects in acute pancreatitis (28). Although the role of renalase and PMCA4b on inflammatory cell populations needs investigation, renalase could likely reduce pancreatitis injury by decreasing pancreatic acinar cell inflammatory injury, and possibly other critical tissue targets.

Our study does have limitations. First, studies were performed in only two mouse models; one was selected to reflect post-ERCP pancreatitis and the other for severe pancreatitis. Although the relevance of rodent pancreatitis models to human disease remains unclear, other models that may share mechanisms with clinical disease, such as ethanol feeding with endotoxin (29), will be examined in the near future. The doses of renalase peptide selected for this study were based on our preliminary studies in pancreatitis, suggesting maximal benefit at the 20 mg/kg doses given here. Future studies will examine the full concentration dependence of the renalase peptides and their impact on a broader range of acute pancreatitis responses, including lung and renal injury and cytokine and chemokine responses.

In summary, we find that renalase peptides containing amino acids 220–239 of human renalase-1 retain the prosurvival and anti-inflammatory activities of the full-length molecule and significantly reduce inflammation and injury in models of severe acute pancreatitis and post-ERCP pancreatitis. These peptides similarly reduced severity in both male and female mice and were effective therapeutically in the severe pancreatitis model. These data suggest that peptides based on human renalase 1 aa 220–239 are as effective as the full-length molecule in reducing injury in experimental pancreatitis and could be attractive agents for therapeutic development.

## DATA AVAILABILITY

Original data is available from the corresponding author upon request.

## GRANTS

Research support for studies of the severe cerulein pancreatitis model was from Congressionally Directed Medical Research Programs under Grant No. PR220457 (to F.S.G. and G.V.D.), and Veterans Affairs Merit Award BX003250 (to F.S.G.). Research support for studies of the post-ERCP model was from National Natural Science Foundation of China under Grant No. 82122010, the National High Level Hospital Clinical Research Funding under Grant No. 2022-PUMCH-E-003, the CAMS Innovation Fund for Medical Science under Grant No. 2023-I2M-3-001 (to L.W.).

## DISCLOSURES

G.V.D. is a named inventor of patents issued related to renalase’s discovery and therapeutic use. Renalase is licensed to Bessor Pharma, and Gary V. Desir holds an equity position in Bessor and its subsidiary Personal Therapeutics. None of the other authors has any conflicts of interest, financial or otherwise, to disclose.

## AUTHOR CONTRIBUTIONS

G.V.D., L.W., and F.S.G. conceived and designed research; T.R.K., X.G., C.A.S., and X.G. performed experiments; T.R.K., X.G., X.G., G.V.D., L.W., and F.S.G. analyzed data; T.R.K., C.A.S., X.G., L.W., and F.S.G. interpreted results of experiments; T.R.K., X.G., and X.G. prepared figures; T.R.K., X.G., and L.W. drafted manuscript; G.V.D., L.W., and F.S.G. edited and revised manuscript; T.R.K., X.G., C.A.S., G.V.D., L.W., and F.S.G. approved final version of manuscript.
